# Fibroblast growth factor 2 accelerates the epithelial–mesenchymal transition in keratinocytes during wound healing process

**DOI:** 10.1038/s41598-020-75584-7

**Published:** 2020-10-29

**Authors:** Yuta Koike, Mariko Yozaki, Atsushi Utani, Hiroyuki Murota

**Affiliations:** Department of Dermatology, Nagasaki University Graduate School of Biomedical Sciences, Nagasaki, Japan

**Keywords:** Experimental models of disease, Acute inflammation, Skin diseases

## Abstract

In the wound healing process, the morphology of keratinocytes at the wound edge temporarily changes to a spindle morphology, which is thought to occur due to an epithelial–mesenchymal transition (EMT). Fibroblast growth factor (FGF) 2, also called basic FGF, has the potential to accelerate wound closure by activating vascular endothelial cells and fibroblasts. We examined the effects of FGF2 on keratinocyte morphology and EMT in wounded skin. Histological examination of murine wounds treated with FGF2 revealed that wound edge keratinocytes formed thickened and multilayered epithelia. In addition, we detected wound edge keratinocytes migrating individually toward the wound center. These migrating keratinocytes exhibited not only spindle morphology but also down-regulated E-cadherin and up-regulated vimentin expression, which is characteristic of EMT. In FGF2-treated wounds, a PCR array revealed the upregulation of genes related to EMT, including transforming growth factor (TGF) signaling. Further, FGF2-treated wound edge keratinocytes expressed EMT-associated transcription factors, including Snai2, and showed translocation of β-catenin from the cell membrane to the cytoplasm/nucleus. However, in vitro examination of keratinocytes revealed that FGF2 alone did not activate EMT in keratinocytes, but that FGF2 might promote EMT in combination with TGFβ1. These findings suggest that FGF2 treatment of wounds could promote keratinocyte EMT, accelerating wound closure.

## Introduction

The wound healing process generally consists of incorporated and overlapping stages of hemostasis, inflammation tissue formation and remodeling of injured tissue^1,2^. These steps involve the coordinated efforts of several cell types, including keratinocytes, fibroblasts, endothelial cells, macrophages and platelets^3^. Reepithelization is a phenomenon in the new tissue formation process in which keratinocytes migrate from the wound edge to the wound center on regenerated granulation tissue, driven by growth factors and cytokines released from sites of injury. In both in vitro and in vivo models, wound edge keratinocytes prepare for migration to the wound center through molecular, morphological, cytoskeletal and adhesive changes^4–6^. In this process, keratinocytes shift from a polarized cuboidal morphology to a more spindle morphology with an extended cytoplasm in order to migrate and close wounds rapidly, which is thought to be an epithelial–mesenchymal transition (EMT).

EMT is a biological process that allows tightly organized epithelial cells to assume a motile mesenchymal phenotype^7,8^. EMT is associated with embryonic and organ development, tissue regeneration and fibrosis, cancer progression and metastasis, and wound healing process^7,9,10^. Although the phenomenon of EMT has not been definitively characterized, EMT is generally accepted as occurring when epithelial cells lose E-cadherin expression and gain expression of mesenchymal cell components such as vimentin and N-cadherin^11^. In wound healing-associated EMT, the spatiotemporally controlled process of keratinocyte formation, in which keratinocytes can transition into a mesenchymal state and revert to an epithelial state, is proposed to be partial EMT and essential for reepithelization system^12,13^. EMT in the context of cancer progression is well studied, and is often compared to EMT in the context of wound healing, mainly with respect to the molecular changes and cell plasticity common to both processes^14^. Cancer progression-related EMT also includes a niche of intermediate states between the epithelium and mesenchyme^15,16^ like partial EMT on wound healing. Snai2, which belongs to the Snai1 superfamily of zinc finger transcriptional repressors, is the most investigated molecule in wound healing EMT, and is thought to be the main EMT-inducing transcription factor in cutaneous wound healing^6,12,13,17,18^.

Fibroblast growth factor (FGF) 2, also called basic FGF, is a member of a large FGF family of structurally related proteins that bind heparin sulfate and modulate the growth, differentiation, migration and survival of a wide variety of cell types^19^. FGF2 strongly activates not only fibroblasts but also other mesoderm-derived cells, including vascular endothelial and smooth muscle cells, osteoblasts and chondrocytes^20^. Administration of recombinant FGF2 to skin wounds accelerates acute and chronic wound healing^21–23^. Therefore, topical recombinant FGF2 has been approved in Japan for the treatment of skin ulcer since 2001. Local FGF2 administration also has an anti-fibrotic effect for the wound to antagonize myofibroblast differentiation and dampen fibrosis which has been evidenced by decreased SMA^24^ and fibronection in the wound tissue^25^. In addition, FGF2 is considered to accelerate reepithelization^26^, which is especially prevalent in an epidermis-defect wound model^27^. However, most in vitro studies have reported that FGF2 is less active in keratinocytes that are derived from primitive ectoderm, relative to mesoderm-derived cells^28^.

In the present study, we detected wound edge migrating and spindle keratinocytes in a mouse cutaneous wound model, which were more prevalent in FGF2-treated wounds. We tested the hypothesis that local wound FGF2 treatment would cause wound edge keratinocytes to undergo EMT, facilitating rapid migration on the wound bed to speed wound closure. Further, we characterized FGF2-induced temporal changes in keratinocytes, focusing on the molecular mechanisms of EMT.

## Results

### FGF2 accelerated wound healing, accompanied by individually migrating keratinocytes

**Figure 1:**
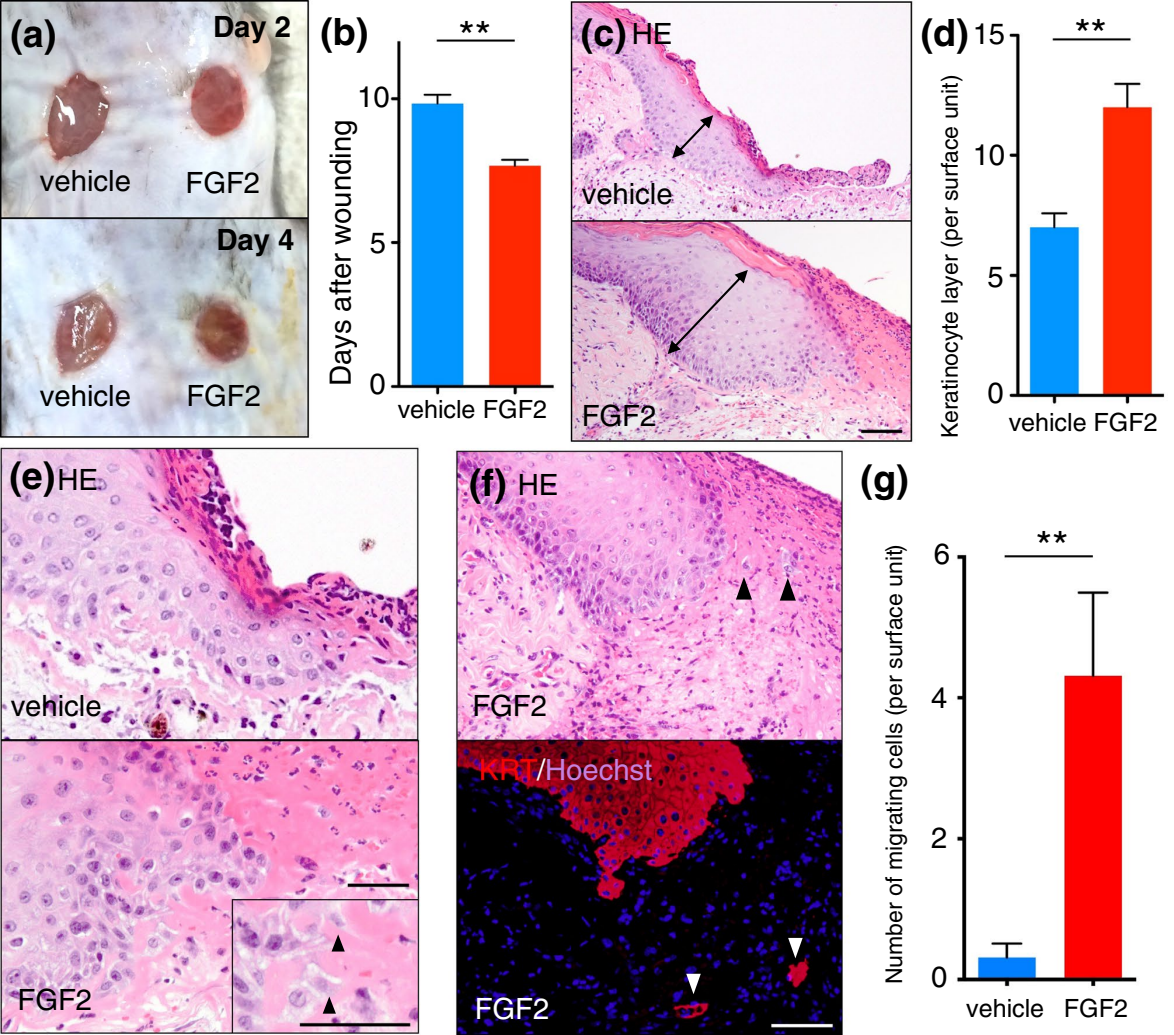
FGF2 accelerated wound healing, epidermal hypertrophy and keratinocytes migration at wound edge. (**a**) Murine wounds were created on the dorsal skin using 6 mm punch, and FGF2-treated wounds were significantly smaller on day 4. (b) Days required for wound closure were significantly decreased by FGF2 treatment. (**c**,**d**) FGF2-treated wounds demonstrated a more thickened epidermis (double arrow) than vehicle-treated wounds. Scale bar: 40 μm. (**e**) At the wound edge, spindle-shaped keratinocytes (arrow heads) were observed in FGF2-treated wounds (higher magnification images at the corner). Scale bar: 20 μm. (**f**) Cells migrating in fibrin clots (arrow heads) were confirmed to be keratinocytes by immunofluorescent staining with anti-pan keratin antibody. Scale bar: 40 μm. (**g**) The number of migrating keratinocytes was significantly higher in FGF2-treated wounds than in control wounds. Bar graphs are presented with the mean values ± standard error. FGF2: fibroblast growth factor 2, HE: hematoxylin and eosin staining. **p < 0.01, Mann–Whitney *U* test.

To investigate the effects of FGF2 on cutaneous wound healing, 6 mm biopsy punch wounds were generated on the dorsal regions of C57BL/6 mice and wound healing was observed. On days 2 and 4, FGF2-treated wounds closed more rapidly than vehicle-treated wounds (Fig. 1a). The average period required for wound closure was significantly shorter in the FGF2-treated group (7.8 ± 0.2 days) than in the control group (9.8 ± 0.3 days, *p* < 0.01; Fig. 1b). In addition, histological examination 4 days after wounding revealed thickening on the edge of the epidermal layer in FGF2-treated wounds (Fig. 1c). The numbers of keratinocytes layer of wound edge were increased in FGF2-treated wounds, with an average of 12.5 ± 2.5 keratinocytes in the FGF2-treated group and 7.0 ± 1.5 keratinocytes in the control group (*p* < 0.01; Fig. 1d). At day 4 after induction of skin wounds, histological examination revealed that keratinocytes in FGF2-treated wounds protruded from the surface, and that spindle-shaped keratinocytes migrated from the wound edge into fibrin clots or granulation tissue (Fig. 1e). Immunofluorescence staining with an anti-pan-keratin antibody confirmed the migrating cells to be keratinocytes (Fig. 1f). The number of migrating keratinocytes was significantly higher in the FGF2-treated group (4.3 ± 1.2 cells) than in the control group (0.3 ± 0.2 cells, *p* < 0.01; Fig. 1g). Thus, murine FGF2-treated wounds exhibited rapid wound closure, accompanied by epithelial proliferation at the wound edge. Additionally, the characteristic spindle morphology and individual migration observed in FGF2-treated wounds suggested that wound edge keratinocytes had acquired a mesenchymal phenotype.

### Immunofluorescent evaluation of EMT-related cell morphological components

**Figure 2:**
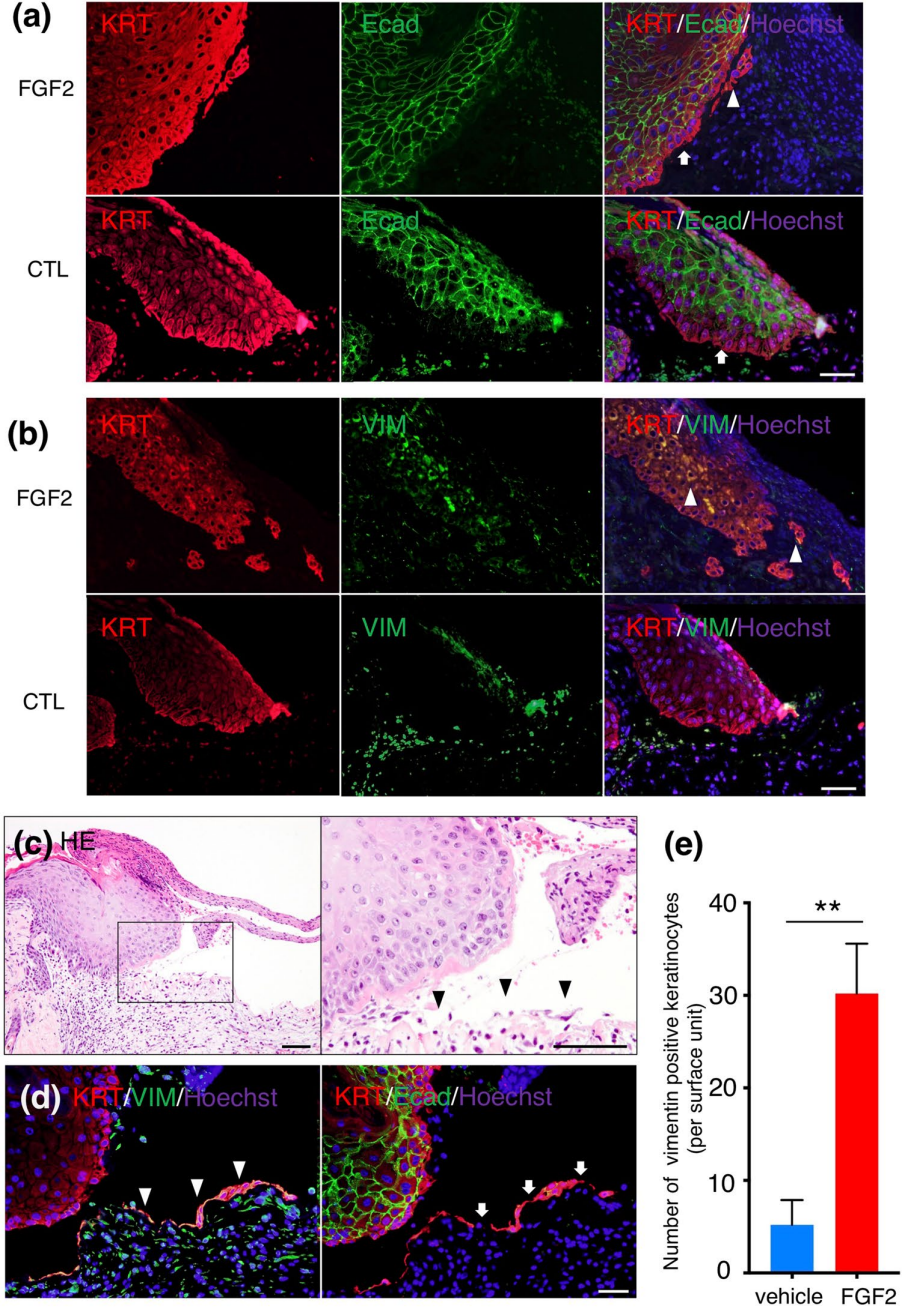
Immunofluorescent staining of wound edges treated with FGF2 or vehicle (control: CTL) after 4 days. (**a**) Cell membrane expression of E-cadherin was reduced in wound edge keratinocytes (arrows) in both FGF2 treated and CTL groups, as well as in keratinocytes away from wound edges in FGF treated group (arrowheads). Scale bar: 20 μm. (**b**) Keratinocytes at the wound edge and during migration in the fibrin clot co-expressed cytokeratin and vimentin (arrowheads) in FGF2 treated group. Scale bar: 20 μm. (**c**) Among HE sections of FGF2-treated wounds, spindle-shaped monolayer keratinocytes (arrowheads) were detected on granulation tissue Scale bar: 40 μm (**d**) and showed were strongly positive for vimentin as well as cytokeratin (arrowheads). In addition, cell membrane E-cadherin was nearly undetectable (arrows). Scale bar: 20 μm (**e**) The number of vimentin-positive wound edge and migrating keratinocytes at the FGF2 treated condition was significantly more than vehicle-treated wounds. Bar graph is presented with the mean values ± standard error. KRT: pan-keratin, Ecad: E-cadherin, VIM: vimentin. **p < 0.01, Mann–Whitney *U* test.

To determine if the observed individually migrating cells had acquired mesenchymal cell characteristics, we immunostained FGF2-treated wounds 4 days after wounding. Cell membrane E-cadherin expression was reduced in wound edge keratinocytes both in FGF2 treated wounds and in vehicle-treated wounds. The down-regulated E-cadherin was also seen in keratinocytes detached from the wound edge in the fibrin clot (Fig. 2a). Interestingly, the wound edge and individually migrating keratinocytes in the FGF2-treated wound co-expressed the intermediate filaments cytokeratin and vimentin (Fig. 2b). Among sections of FGF2-treated wounds on day 4, we detected spindle-shaped monolayer keratinocytes on the portion of granulation tissue referred to as the epithelial tongue (Fig. 2c). These migrating keratinocytes were strongly positive for vimentin and cytokeratin. In addition, cell membrane E-cadherin was nearly undetectable (Fig. 2d). The number of vimentin and cytokeratin co-expression cells in the wound edge and migrating keratinocytes were significantly higher in FGF2 treated wounds (30.2 ± 5.3 cells) than in the control group (5.2 ± 2.6 cells, *p* < 0.01; Fig. 2e). Thus, these findings suggested that wound edge and migrating keratinocytes decreased E-cadherin regardless of the FGF2 application or not. In addition, those keratinocytes observed in FGF2-treated wounds acquired typical morphological and molecular features of EMT, which were characterized by not only decreased E-cadherin but also increased vimentin.

### PCR array analysis of wound tissue EMT-associated molecules

**Figure 3:**
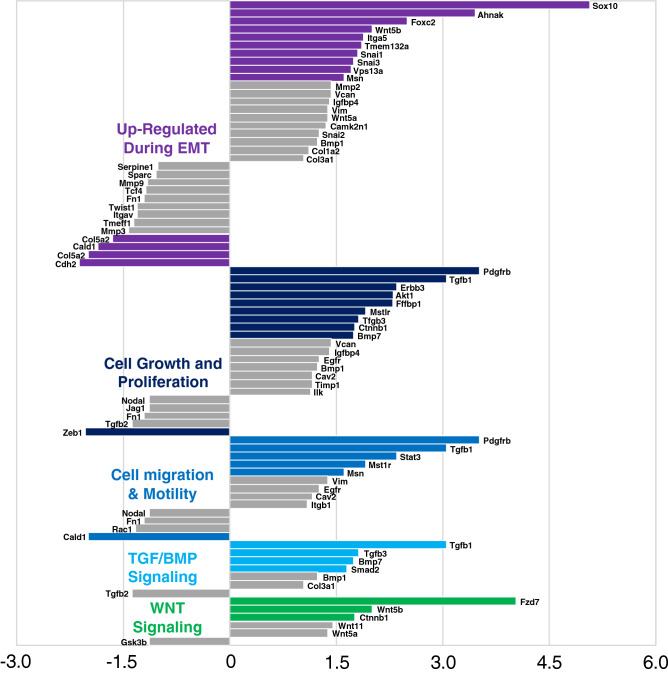
Tissue PCR array analysis about EMT associated components in murine wounds. Eighty-four EMT associated components were examined in RNA extracted from whole wounds at day 4 treated with vehicle or FGF2 (n = 3/group). Genes were categorized into five groups, including genes typically upregulated during EMT, cell growth and proliferation genes, cell migration and motility genes, genes related to TGF/bone morphogenetic protein (BMP) signaling, and genes related to Wnt signaling. Genes indicated with colored bars were up or down-regulated > 1.5 fold change.

**Figure 4:**
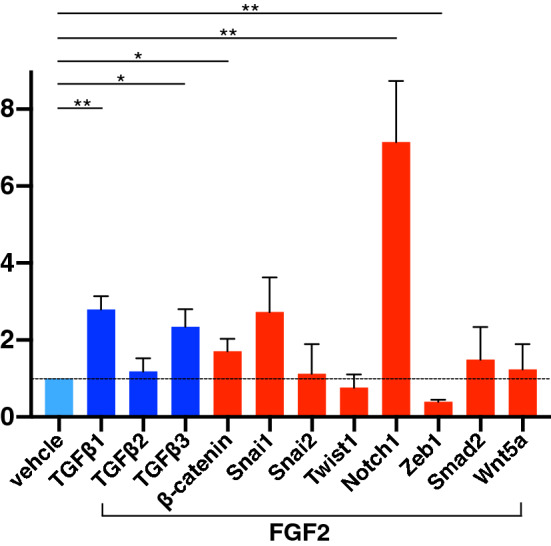
Representative EMT-associated upregulated genes in PCR array analysis. Individual genes of the FGF2 group were normalized by the same gene of vehicle group respectively. TGFβ1 (2.79 fold: *p* < 0.01), TGFβ2 (1.18 fold), TGFβ3 (2.34 fold: *p* < 0.05), β-catenin (1.70 fold: *p* < 0.05) and Notch1 (7.14 fold: *p* < 0.01) were up-regulated and Zeb1 (0.39 fold: *p* < 0.01) was down-regulated significantly in comparison with vehicle-treated samples. Bar graph is presented with the mean values ± standard error. *p < 0.05, **p < 0.01, Mann–Whitney *U* test.

A tissue PCR array of 84 EMT-associated components (See Supplementary Data. S2 online) was performed using RNA extracted from full-thickness mouse wounds on day 4, treated with FGF2 (n = 3) or PBS (n = 3). Out of targets, 34 transcripts were at least 1.5-fold upregulated, and 11 were at least 1.5-fold down-regulated in FGF2-treated wounds compared to control wounds. Genes were categorized into five groups, including genes typically upregulated during EMT, cell growth and proliferation genes, cell migration and motility genes, genes related to TGF/bone morphogenetic protein (BMP) signaling, and genes related to Wnt signaling. Of these gene categories, the 34 upregulated genes in the in FGF-treated group included ten genes in the EMT category, nine genes in the cell growth and proliferation category, five genes in the cell migration and motility category, four genes in the TGF/BMP signaling category and three genes in the Wnt signaling category (Fig. 3). Fewer genes in each category were downregulated in the FGF2 group. To confirm the expression of major EMT-associated genes, we selected representative genes that are commonly investigated in the study of EMT from the PCR array data. Individual results from PCR array revealed changes in mRNA levels of TGFβ1 (2.79 ± 0.34 fold: *p* < 0.01), TGFβ2 (1.18 ± 0.34 fold), TGFβ3 (2.34 ± 0.45 fold: *p* < 0.05), β-catenin (1.70 ± 0.32 fold), Snai1 (2.72 ± 0.90 fold), Snai2 (1.12 ± 0.90 fold), Twist1 (0.76 ± 0.34 fold), Notch1 (7.14 ± 1.59 fold: *p* < 0.01), Zeb1 (0.39 ± 0.04 fold: *p* < 0.01), Smad2 (1.48 ± 0.84 fold:) and Wnt5a (1.23 ± 0.66 fold) in the FGF2 group relative to control (Fig. 4). Taken together, these findings suggested that EMT associated components, including transcriptional regulators and cytokines in the TGF/BMP and Wnt signaling axes, were activated in FGF2-treated wound tissue. TGFβ1, a potent inducer of EMT, was significantly upregulated in FGF2-treated wound tissue, suggesting that TGFβ1 could be a key regulator of FGF2-mediated EMT enhancement.

### Immunofluorescent staining of EMT-associated transcription factors

**Figure 5:**
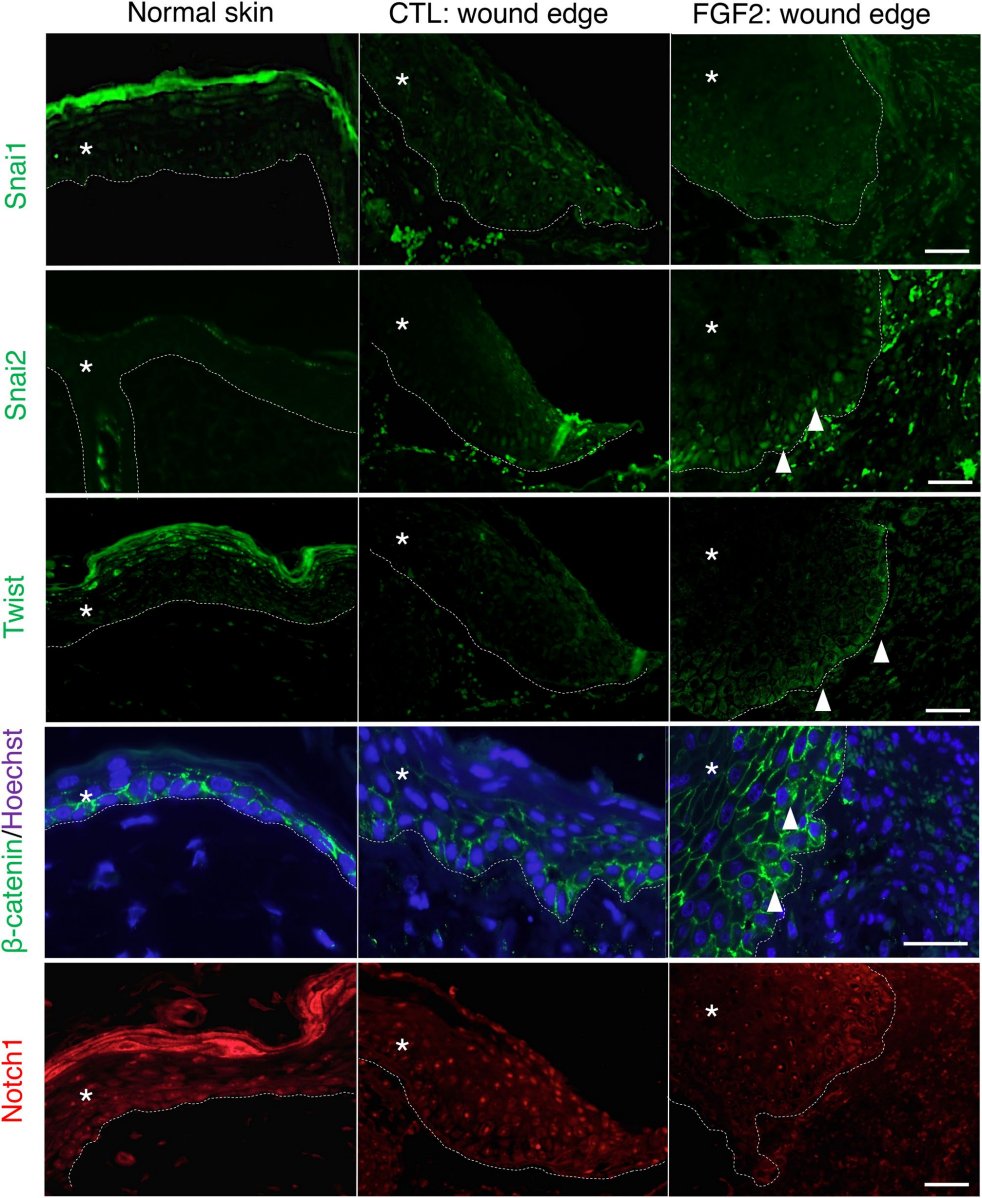
Immunofluorescent staining for EMT associated transcriptional factors was performed on non-injured skin (normal skin) and wound edges treated with vehicle (CTL) and FGF2 4 days after wounding. Snai1 was absent in all normal skin, CTL wounds, and FGF2-treated wounds. Snai2 was robustly expressed in wound edge keratinocytes in FGF2-treated wounds (arrowheads) and faintly expressed in those in control wounds. Twist was expressed in FGF2-treated wounds (arrowheads). β-catenin translocated from the cell membrane to the cytoplasm or nuclei (arrowheads) in the wound tissues, especially in the FGF2-treated group. Notch1 did not differ among samples. Scale bar: 40 μm, *shows epidermis.

We performed tissue immunofluorescent staining of the non-affected epidermis (normal skin), vehicle-treated wound (control), and FGF2-treated wound at day 4 after wounding to evaluate expression and localization of EMT-associated factors at wound edge keratinocytes. As a representative result (Fig. 5), Snai1 was not present in normal skin, control and FGF2 treated-wound. Snai2 was robustly expressed in the nucleus of wound edge keratinocytes in the FGF2 group, faintly expressed in a few keratinocytes in control and not expressed in normal skin. Twist was detected in the cytoplasm of wound edge keratinocytes in the FGF2 group, but almost not detected in the normal skin and in the control group. β-catenin comprises the cell attachment structure of keratinocytes. In FGF2-treated wounds, some wound edge keratinocytes showed activation of β-catenin signaling evidenced by translocation of β-catenin from the cell membrane to the cytoplasm or nucleus, which was slightly seen in the control mock treated group. The immunofluorescent staining pattern of Notch1 did not differ among normal epidermis, control wounds and FGF2-treated wounds. Taken together, these findings suggest that keratinocyte EMT in FGF2-treated wounds might be regulated by transcription factors such as Snai2, Twist and β-catenin.

### In vitro assay of cultured keratinocytes treated with FGF2 and TGFβ1

**Figure 6:**
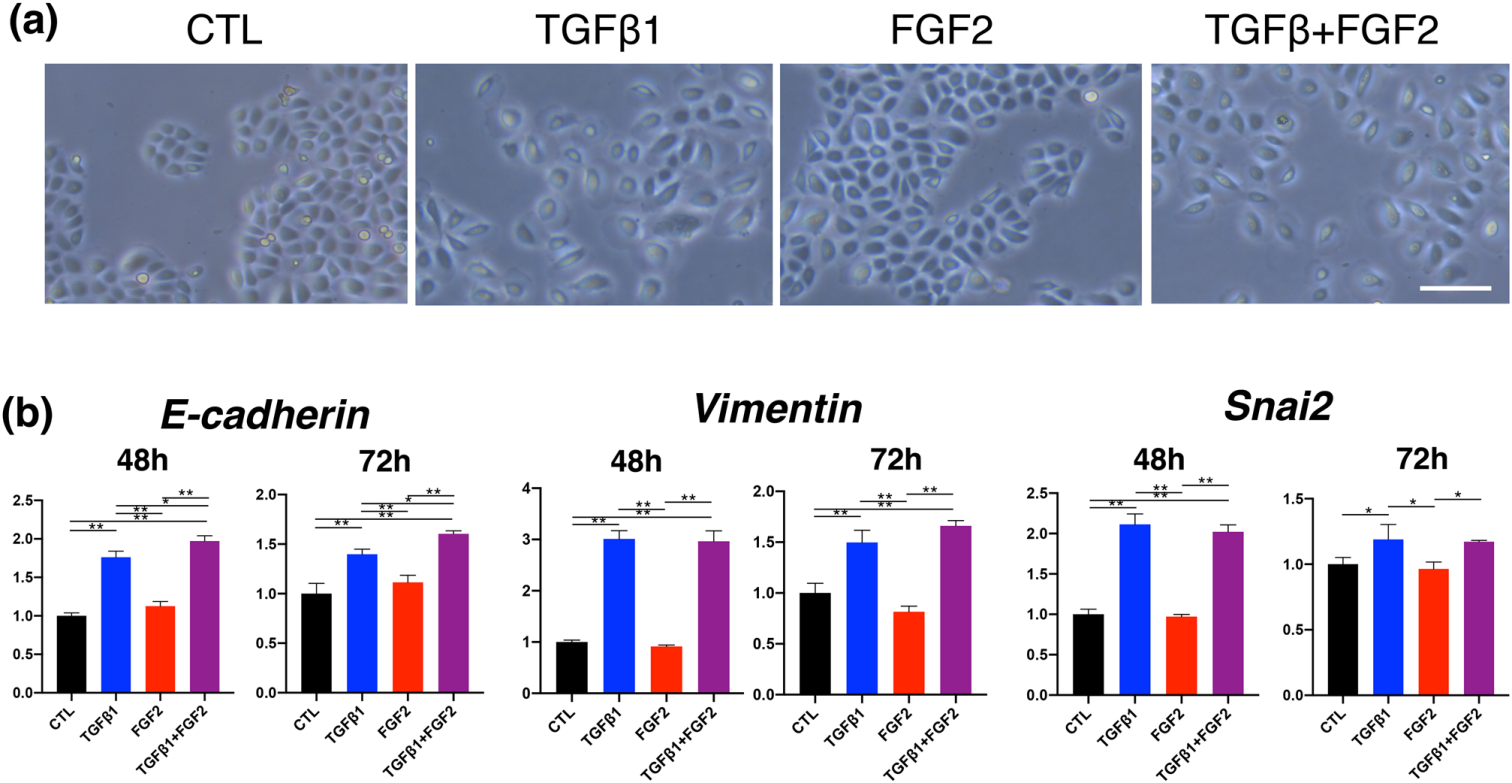
In vitro experiment using normal human epidermal keratinocytes (NHEKs) to compare among normal culture condition (CTL), TGFβ1 solo-stimulation, FGF2 solo-stimulation, and TGFβ1 and FGF2 co-stimulation (TGFβ1 + FGF2). (**a**) After 48 h of stimulation, NHEKs with TGFβ1 and TGFβ1 + FGF2 showed disseminated proliferation in contrast to the cobblestone pattern of those with normal medium and FGF2 treated condition. Scale bar: 20 μm (**b**) realtime RT-PCR analysis of NHEKs. The Y-axis represents the relative ratio normalized by CTL. mRNA expressions of E-cadherin, vimentin, and Snai2 at 48 and 72 h stimulation were highly and significantly upregulated in TGFβ1 and TGFβ1/FGF2. Solo treatment with FGF2 affected little in expressions of EMT-related transcription factors in NHEKs. Bar graphs are presented with the mean values ± standard error. *p < 0.05, **p < 0.01, ANOVA with a post hoc Tukey examination.

**Figure 7:**
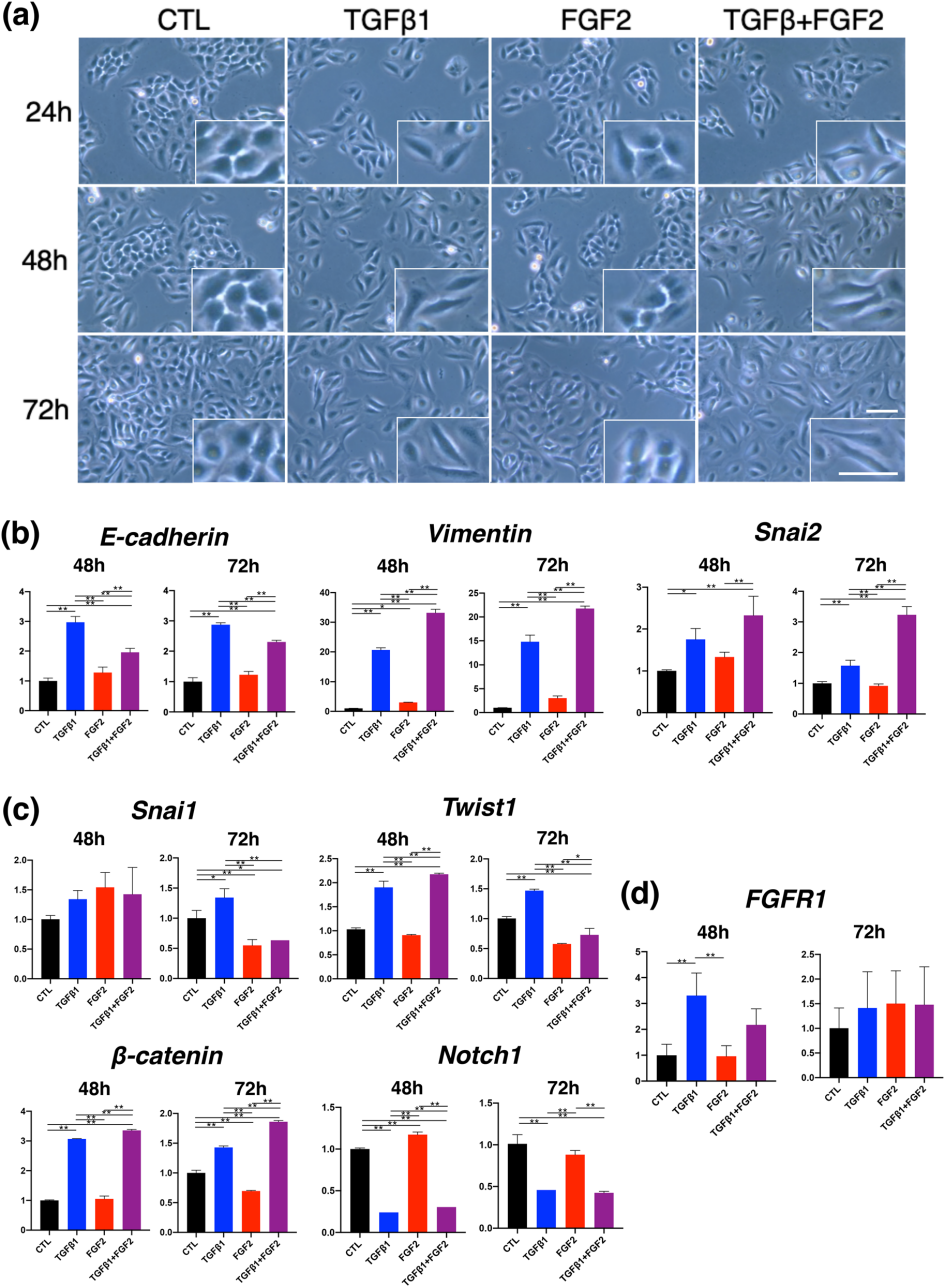
In vitro experiment using HaCaT cells to compare among normal culture condition, TGFβ1 solo-stimulation, FGF2 solo-stimulation, and TGFβ1 and FGF2 co-stimulation (TGFβ1 + FGF2). (**a**) HaCaT cells gradually showed not only the disseminated pattern but also spindle morphology with TGFβ1 and TGFβ1 + FGF2 treatment in the time course of cell culture from 24 to 72 h. Higher magnification images are at the corners. Scale bar: 20 μm (**b**) Realtime RT-PCR analysis of HaCaT cells. The Y-axis represents the relative ratio compared with CTL. mRNA expression of E-cadherin, vimentin and Snai2 was highly and significantly upregulated in TGFβ1. Vimentin and Snai2 were further upregulated by TGFβ1 + FGF2 co-treatment than TGFβ1 solo treatment especially at the time point of 72 h. (**c**) mRNA expression of Snai1 increased with TGFβ1 but suppressed with FGF2 and TGFβ1 + FGF2 at 72 h. Twist1 was increased with TGFβ1 at 48 and 72 h, and TGFβ1 + FGF2 at 48 h. β-catenin was similarly upregulated by TGFβ1 and TGFβ1 + FGF2 treatment at both time points. Notch1 was significantly suppressed by TGFβ1 and TGFβ1 + FGF2 treatment. Solo treatment with FGF2 affected little in expressions of EMT-related transcription factors in HaCaT cells. (**d**) Transcription of FGFR1 in HaCaT cells showed a significant increase with TGFβ1 in comparison with control and FGF2 treatment at 48 h. Bar graphs are presented with the mean values ± standard error. *p < 0.05, **p < 0.01, ANOVA with a post hoc Tukey examination.

In wounds treated with FGF2, we identified TGFβ1 mRNA upregulation in the whole tissue extract and upregulation of EMT-associated transcription factors in wound edge keratinocytes. As TGFβ1 is a major inducer of EMT^12^, we next used an in vitro assay, in which normal human epidermal keratinocytes (NHEKs) and a keratinocyte cell line were treated with TGFβ1 and FGF2 solo-stimulation and TGFβ1 and FGF2 co-stimulation (TGFβ1 + FGF2) to determine whether FGF2 contributed to the EMT on keratinocyte. In the experiment using NHEKs, TGFβ1 and TGFβ1 + FGF2 stimulation showed disseminated proliferation of cells that implied EMT in contrast to the cobblestone pattern of control and FGF2 treated cells 48 h after stimulation (Fig. 6a). mRNA expression of E-cadherin, vimentin, and Snai2 was highly and significantly upregulated in TGFβ1 and TGFβ1/FGF2. Whereas treatment of cells with FGF2 alone did not alter the expression of E-cadherin, vimentin, and Snai2. TGFβ1 alone significantly upregulates the mRNA of these genes, however, co-treatment of keratinocytes with TGFβ1 and FGF2 further enhances the expression of only E-cadherin (Fig. 6b). A keratinocyte cell line, HaCaT cells, gradually showed not only disseminated pattern but also spindle morphology with TGFβ1 and TGFβ1 + FGF2 treatment in the time course of cell culture (Fig. 7a). In contrast, morphology was unaffected with FGF2 alone. mRNA expression of E-cadherin, vimentin and Snai2 was highly and significantly upregulated in TGFβ1. Vimentin and Snai2 were further upregulated by TGFβ1 + FGF2 co-treatment than TGFβ1 solo treatment especially at the time point of 72 h (Fig. 7b). As these results using HaCaT cells revealed EMT with TGFβ1 and might imply enhancement of the TGFβ1-induced EMT with FGF2, we examined the mRNA expression of other EMT associated molecules (Fig. 7c). Snai1 increased with TGFβ1 but suppressed with FGF2 and TGFβ1 + FGF2 at 72 h. Twist1 was increased with TGFβ1 at 48 and 72 h, and TGFβ1 + FGF2 at 48 h. β-catenin was similarly upregulated by TGFβ1 and TGFβ1 + FGF2 treatment at both time points. Among EMT-inducing transcription factors, Notch1 was significantly suppressed by TGFβ1 treatment and TGFβ1 + FGF2 co-treatment. Treatment with FGF2 alone had little effect on the expression of EMT-related transcription factors in NHEK and HaCaT cells. However, when we see the expression levels of vimentin and Snai2 in TGFβ1-stimulated HaCaT cells with or without FGF2, FGF2 might affect the synergistic effect of these genes under TGFβ1 stimulation. To resolve the mechanism, we measured mRNA of FGFR1 which was one of the FGF2 receptors. As a result, FGFR1 showed a significant increase with TGFβ1 in comparison with control and FGF2 at 48 h in HaCaT cells (Fig. 7d). Taken together, NHEKs and HaCaT cells treated with TGFβ1 showed EMT but not with FGF2 alone. In HaCaT cells, some EMT markers like vimentin and Snai2 were further upregulated by TGFβ1 + FGF2 co-treatment relative to TGFβ1 treatment alone, suggesting that FGF2 might enhance TGFβ1-mediated EMT in keratinocytes via agonizing TGFβ1-upregulated receptors like FGFR1 by expression of FGF2 recognizable receptors such as FGFR1 by TGFβ1.

## Discussion

We investigated the FGF2 function in the wound healing process, focusing specifically on keratinocytes. As reported in several papers before^21,23,26,27,29^, FGF2 treated wound closed faster than vehicle-treated wounds. In the epidermis of wound edge, in addition to epidermal hypertrophy, spindle-shaped and migrating keratinocytes were observed. These characteristic cells revealed to express intermediate filaments, not only cytokeratin but also vimentin which usually express in mesenchymal cells and to reduce their expression of E-cadherin, suggesting an EMT. In order to evaluate the tissue molecular condition in the FGF2 treated wound, we performed a PCR array using the wound tissue which showed that the tissue environment tended to initiate EMT as well as cell growth, proliferation, cell migration and motility, and accelerate TGF/BMP signaling and WNT signaling. EMT associated transcriptional factors such as Snai2, Twist and β-catenin were also expressed in the wound edge keratinocytes. In in vitro examination, FGF2 treatment showed no influence on NHEKs and HaCaT cells, but FGF2 might have additional EMT effects to cells when co-stimulating with TGFβ.

In partial epidermal EMT during wound healing, the cell–cell adhesion of keratinocytes is characterized by diminished desmosome components and adherens junction components, such as E-cadherin^5,6,30,31^. In contrast to carcinomas, upregulation of vimentin in wound healing-related EMT is still unclear^13^. Only one report demonstrated vimentin expression in migrating epithelial tongues of acute wounds and hypertrophic scars^32^. The present study identified vimentin expression in FGF2-treated wounds, especially in keratinocytes observed on the epithelial tongue, indicating that vimentin expression in keratinocytes during wound healing EMT could require specific conditions, such as tissue activation with FGF2. A study has revealed that vimentin-deficient wounds exhibited loss of EMT-like keratinocyte activation, limited keratinization and slow reepithelization^33^, demonstrating the importance of vimentin-positive keratinocytes in the reepithelization process.

Among EMT-associated transcription factors, several reports support the functional involvement of Snai2 in cutaneous wound healing^6,17,18^. In the present study, Snai2 expression was clearly observed in wound edge keratinocytes treated with FGF2 in vivo. Additionally, TGFβ1 induced Snai2 mRNA expression by which was significantly upregulated by co-treatment with FGF2 in HaCaT cells. A prior study identified that *Snai2-*null mice presented non-healing cutaneous ulcers in response to ultraviolet radiation^6,34,35^. Upregulation of Snai2 in the nuclei of keratinocytes at the edges of FGF2-treated wounds^36,37^ supported the importance of Snai2 in keratinocyte migration and tissue regeneration. Other EMT-associated transcription factors such as Twist^38^ and β-catenin^36,37^ have been reported to express in keratinocytes at wound-healing process, as well as in tumor cells as hallmarks of EMT^14^.

The Snai1 family, including Snai1 and Snai2, regulates EMT associated gene transcription downstream of TGF signaling^18,39^. FGF2-treated wounds exhibited 3.05-fold upregulation of TGFβ1 in comparison to vehicle-treated wounds. This result indicated that TGFβ1 could have provoked EMT as a messenger of FGF2 in the wound tissue. Translocation of β-catenin from the cell membrane to the cytoplasm or nucleus was observed in the wound edge keratinocytes of wounds treated with FGF2. E-cadherin not only connects keratinocyte membranes, but also sequesters β-catenin to the cell membrane to suppress Wnt–β-catenin signaling, which regulates key migration events of EMT^40^. In keratinocytes, this phenomenon is thought to participate in the wound healing process^41^. Notch signaling has various functions, including epidermal differentiation^41^ and EMT^11^. Our PCR array results revealed greater expression of Notch1 mRNA in FGF2-treated wound tissue than in control wound tissue, however Notch1 was only marginally induced in wound edge keratinocytes in either group and also down-regulated in in vitro HaCaT model treated with TGFβ1 and TGFβ1 + FGF2. Notch1 is essential for epidermal proliferation and differentiation^42^. A recent study revealed that Notch1 expression is down-regulated in epidermal keratinocytes at the regeneration phase in the wound healing process^43^. From these facts, Notch1 would contribute to suppress epidermal differentiation but not influence the EMT in wound edge keratinocytes even when stimulating with topical FGF2. The significant up-regulation of Notch1 at the PCR array data in FGF2 treated wound tissue might be due to overall activation of cells especially in the regenerated dermis. Taken together, our results suggest that when FGF2 is applied to wounds during the acute phase, increased TGFβ1 may stimulate EMT signaling in wound edge keratinocytes, upregulating EMT-associated transcription factors such as Snai2. Finally, EMT, which is characterized by diminished E-cadherin and expression of vimentin, could occur, supporting wound healing.

Although FGF2 accelerates reepithelization in the wound healing process^26,27^, the direct effects of FGF2 on epidermal keratinocytes remain controversial. A small number of studies have suggested that direct FGF2 stimulation activates keratinocytes. In vitro studies using NHEKs demonstrated that FGF2 could stimulate keratinocyte proliferation^44^. *Nakamizo* et al. demonstrated FGF2 promoted NHEKs proliferation, which was inversely dependent on cell density, as cells in high-density culture exhibited decreased FGF receptor expression^45^. In the present study, we demonstrated that FGF2 treatment alone had little impact on EMT and cell morphology in NHEKs and HaCaT cells. Intriguingly, our results on HaCaT cells also suggested that TGFβ1/FGF2 co-treatment upregulated EMT-associated genes more robustly than TGFβ1 treatment alone. *Shirakihara* et al. suggested that TGFβ and FGF2 cooperatively initiate EMT using NMuMG cells, a mouse mammary epithelial cell line. When NMuMG cells were co-stimulated with TGFβ and FGF2, TGFβ regulated isoform switching of FGF receptors from FGFR2IIIb into a FGF2-recognizable FGF receptor, FGFR1IIIc. Consequently, FGF2 co-treatment increased the migratory capacity of NMuMG cells, and induced morphological changes consistent with EMT relative to TGFβ treatment alone^46^. Our HaCaT model also might suggest a similar mechanism, by which TGFβ might initiate FGF receptor isoform switching into FGFR1 to allow FGF2 recognition, further promoting EMT.

In the present study, several limitations exist. In the in vivo wound healing model mainly documented in Fig. 1, additional factors modulating the acceleration of wound healing should be considered. Skin contraction resulting in faster healing, with temporarily increased myofibroblast is the main player^47^. TGFβ is known to increase differentiation of fibroblasts into myofibroblasts^48,49^, and thus, local administration of FGF2 which induce TGFβ mRNA in the tissue may indirectly reduce time to healing. On the other hand, local administration of FGF2 is also reported to suppress differentiation of fibroblasts to myofibroblasts in vivo^50,51^. Taken together, topical administration of FGF2 was revealed to shorten the period of wound healing despite suppression of myofibroblast differentiation. The mechanism responsible for FGF2 accelerating wound healing without skin contraction can be speculated to include neovascularization, modulated collagen and matrix deposition, as well as keratinocyte EMT. Various cell types may participate in these processes, but the current study focused on wound edge keratinocytes, representing a difficult-to-quantify site, both in morphology and molecular expression.

In summary, the results obtained in the present study suggest that topical application of FGF2 on cutaneous wound upregulates tissue mRNA of TGFβ1 and other EMT associated molecules, accelerates EMT of wound edge keratinocytes, and might contribute to reepithelization during new tissue formation.

## Materials and methods

### Mouse wound healing experiment

All experiments were approved by the committee for Animal Experimentation of Nagasaki University Graduate School of Biomedical Sciences (approval number 1710261420). All methods were carried out in accordance with relevant guidelines and regulations. Male C57BL6/J mice, 8–12 weeks old, were used for experiments and analysis. The dorsal skin was shaved and and two full thickness excisional wounds were created with a 6 mm sterile biopsy punch on the left and right dorsal skin. Recombinant FGF2 (trafermin; Kaken, Japan) diluted to 100 μg/ml (approved concentration for skin ulcer on human^29^) with phosphate buffered saline (PBS) was prepared. The FGF2 solution (50 μl) was dropped on the right wounds, and PBS (50 μl) was dropped on the left wounds as a control, and then covered with film dressing. For the following days, daily treatment with 50 μl PBS or FGF2 solution and dressing changes were performed. The day of macroscopic wound closure was defined as the first day with no exudate fluid and no scab (n = 6, respectively).

### Histology

Skin samples day 4 after wounding were obtained from seven murine and fixed in formalin and paraffin-embedded. Sections were stained with hematoxylin and eosin staining. Morphologic characteristics of wound samples were assessed by light microscopy. We chose one section whose both sides of epidermal edges had been clearly found from individual samples and counted numbers of cell layers at the most thickened site and migrating keratinocytes of the bilateral epidermis wound edge (n = 14, individually).

### Immunofluorescence analysis

For immunofluorescence analysis of paraffin sections, four samples were deparaffinized and antigens were retrieved with autoclave 121 °C 3 min, in 10 mM Sodium Citrate Buffer (pH6.0). After washing, samples were blocked with 10% fetal bovine serum (FBS)/PBS at room temperature for 30 min. Primary antibodies (See Supplementary Data. S1 online) were used at 1:50–200 dilutions in 10%FBS/PBS and incubated overnight at 4 °C. After washing, secondary antibodies (goat anti-mouse or rabbit IgG (H + L), Alexa Fluor 488-, 546- or 594-conjugated; Thermo Fisher Scientific Inc. US), 1:100 diluted in 10% FBS/PBS, were used with or without Hoechst 33342 (Invitrogen, USA) for nuclear staining at 37 °C for 30 min. After washing and mounting, sections were observed with a fluorescence microscope. For the detection of vimentin-positive keratinocytes, we chose one section whose both sides of epidermal edges had been clearly found from three individual samples and counted numbers of vimentin-positive keratinocytes from the bilateral epidermis wound edge (n = 6, individually).

### Tissue PCR array

Around the biopsy punch wounds, a 10 mm square of full-thickness tissues of FGF2 treated wounds and vehicle-treated wounds were removed (n = 3, respectively). Tissue samples from mouse wounds were immediately treated with RNAlater (Qiagen, Germany). Total tissue RNA was subsequently treated with DNase I (Qiagen) and further purified using a RNeasy Mini Kit (Qiagen). A total of 1 μg of RNA was purified as per the manufacturer’s protocol then reverse transcribed using a First Strand Synthesis Kit (Qiagen) per manufacturer’s instructions, and subsequently loaded onto an EMT-associated molecule RT^2^ profiler array according to the manufacturer’s instructions (Qiagen). Fold change was calculated by determining the ratio of mRNA level to control values using the Δ threshold cycle (Ct) method (2^−ΔΔCt^).

### Cell culture

NHEKs were obtained from Lonza (Basel, Switzerland) and cultured in keratinocyte growth medium supplemented with bovine pituitary extract, recombinant epidermal growth factor, insulin, hydrocortisone, transferrin, and epinephrine (KGM-Gold Keratinocyte Growth Medium BulletKit, Lonza) and grown in a humidified 5% CO2 atmosphere at 37 °C. For experiments, 0.1 × 10^6^ cells/well (6-well plate) were seeded and grown in the above culture conditions for 24 h to allow cell attachment. Human immortalized keratinocytes (HaCaT cells) were maintained in Dulbecco’s modified eagle medium (Nissui, Japan) supplemented with 10% fetal bovine serum, 50 units/ml penicillin G and 50 ug/ml streptomycin and grown in a humidified 5% CO2 atmosphere at 37 °C. For experiments, 0.3 × 10^6^ cells/well (6-well plate) were seeded and grown in the above culture conditions for 24 h to allow cell attachment, after which the cells were serum-starved for another 24 h. The NHEKs and HaCaT cells were then cultured in the absence or presence of recombinant transforming growth factor (TGF) β1 (2 ng/ml; R&D systems, USA) and recombinant FGF2 (30 ng/ml). Concentrations of recombinant cytokines were determined by referring to previous experiments about FGF2^46,52^ and TGFβ1^53,54^.

### Real-time reverse transcription PCR

Total HaCaT cell RNA was isolated using a RNeasy kit (Qiagen). Total RNA from each sample was reverse-transcribed into cDNA and analyzed with primers (See Supplementary Data. S1 online) and an ABI Prism 7000 sequence detector (Applied Biosystems, US) using a real-time PCR quantification method according to the manufacturer’s instructions and description in the past report^55^. Sequence-specific primers and probes were designed by pre-developed TaqMan assay reagents or Assay-On-Demand (Applied Biosystems). Relative expression of real-time PCR products was determined using the ΔΔ*C*_T_ method to compare target gene and GAPDH mRNA expression.

### Statistical analyses

The results are given as means ± standard error of the mean. Statistical analyses were performed using GraphPad Prism 8 (GraphPad, San Diego, CA, USA). When two groups were analyzed, the data were performed using Mann–Whitney *U* test. When three or more groups were analyzed, the data were analyzed by ANOVA with a post hoc Tukey examination. *p* < 0.05 was considered statistically significant.

## Supplementary information

Supplementary Information
