# Supplementary material for: Fibroblast growth factor 2 accelerates the epithelial–mesenchymal transition in keratinocytes during wound healing process
Source: Sci Rep. 2020 Oct 29;10:18545. doi: 10.1038/s41598-020-75584-7 (PMC7596476; doi:10.1038/s41598-020-75584-7)
Supplement: Supplementary file 1 — Supplementary Information [file 41598_2020_75584_MOESM1_ESM.doc]

**Supplementary information**

Title of manuscript:

**Fibroblast growth factor 2 accelerates the epithelial-mesenchymal transition in keratinocytes during wound healing process**

Names of authors:

**Yuta Koike, Mariko Yozaki, Atsushi Utani, Hiroyuki Murota**

**Supple 1.**

**Primary antibodies**

anti-pan keratin (GP14, PROGEN, Germany), E-cadherin (#3195, Cell signaling), Vimentin (#3932, Cell Signaling Technology Japan, Japan), Snail (ab63371, Abcam), Slug (#9585, Cell Signaling Technology), Twist (ab50887, Abcam, UK), β-catenin (#9587, Cell Signaling Technology) and Notch1 (ab8925, Abcam)

**Primers**

*Cdh1* (Hs01023895_m1, Applied Biosystems, Foster City, CA), *Vim* (Hs00958111_m1, Applied Biosystems), *Ctnnb* (Hs00355045_m1, Applied Biosystems), *Snai1* (Hs00195591_m1, Applied Biosystems), *Snai2* (Hs00161904_m1, Applied Biosystems), *Twist1* (Hs01675818_s1, Applied Biosystems) and *Notch1* (Hs01062014_m1, Applied Biosystems), *FGFR1* (Hs00241111_m1, Applied Biosystems), *GAPDH* (Hs00266705_g1, Applied Biosystems)

**Supple 2.**

List of 84 EMT associated molecules measured in PCR array:

*Ahnak, Akt1, Bmp1, Bmp7, Cald1, Camk2n1, Cav2, Cdh1, Cdh2, Col1a2, Col3a1, Col5a2, Ctnnb1, Dsc2, Dsp, Egfr, Erbb3, Esr1, F11r, Fgfbp1, Fn1, Foxc2, Fzd7, Gng11, Gsc, Gsk3b, Igfbp4, Il1rn, Ilk, Itga5, Itgav, Itgb1, Jag1, Krt14, Krt19, Krt7, Mitf, Mmp2, Mmp3, Mmp9, Msn, Mst1r, Map1b, Nodal, Notch1, Nudt13, Ocln, Pdgfrb, Plek2, Desi1, Ptk2, Ptp4a1, Rac1, Rgs2, Serpine1, Gemin2, Smad2, Snai1, Snai2, Snai3, Sox10, Sparc, Spp1, Stat3, Steap1, Tcf4, Tcf7l1, Tfpi2, Tgfb1, Tgfb2, Tgfb3, Timp1, Tmeff1, Tmem132a, Tspan13, Twist1, Vcan, Vim, Vps13a, Wnt11, Wnt5a, Wnt5b, Zeb1, Zeb2.*
